# Application of a Generative Adversarial Network in Image Reconstruction of Magnetic Induction Tomography

**DOI:** 10.3390/s21113869

**Published:** 2021-06-03

**Authors:** Dan Yang, Jiahua Liu, Yuchen Wang, Bin Xu, Xu Wang

**Affiliations:** 1 Key Laboratory of Data Analytics and Optimization for Smart Industry, Northeastern University, Shenyang 110819, China; 17863904576@163.com (J.L.); Wangxu@mail.neu.edu.cn (X.W.); 2 Key Laboratory of Infrared Optoelectric Materials and Micro-Nano Devices, Shenyang 110819, China; 2070802@stu.neu.edu.cn; 3 College of Information Science and Engineering, Northeastern University, Shenyang 110819, China; 4 College of Computer Science and Engineering, Northeastern University, Shenyang 110819, China; xubin@mail.neu.edu.cn

**Keywords:** magnetic induction tomography, image reconstruction, generative adversarial network, conductivity distribution

## Abstract

Image reconstruction of Magnetic induction tomography (MIT) is an ill-posed problem. The non-linear characteristics lead many difficulties to its solution. In this paper, a method based on a Generative Adversarial Network (GAN) is presented to tackle these barriers. Firstly, the principle of MIT is analyzed. Then the process for finding the global optimum of conductivity distribution is described as a training process, and the GAN model is proposed. Finally, the image was reconstructed by a part of the model (the generator). All datasets are obtained from an eight-channel MIT model by COMSOL Multiphysics software. The voltage measurement samples are used as input to the trained network, and its output is an estimate for image reconstruction of the internal conductivity distribution. The results based on the proposed model and the traditional algorithms were compared, which have shown that average root mean squared error of reconstruction results obtained by the proposed method is 0.090, and the average correlation coefficient with original images is 0.940, better than corresponding indicators of BPNN and Tikhonov regularization algorithms. Accordingly, the GAN algorithm was able to fit the non-linear relationship between input and output, and visual images also show that it solved the usual problems of artifact in traditional algorithm and hot pixels in L2 regularization, which is of great significance for other ill-posed or non-linear problems.

## 1. Introduction

Magnetic induction tomography (MIT) is a kind of electromagnetic imaging technology based on the eddy current testing principle [1]. In general applications, many groups of excitation–detection combination coils are used to obtain induced magnetic field signals near the imaging body [2], and then the conductivity distribution of the range to be measured is calculated by the reconstruction algorithm [3,4]. Because of its non-invasive and non-contact characteristics, MIT is suitable for geological exploration [5], industrial flaw detection [6], impurity detection [7], and medical imaging [8]. The research on it can be roughly divided into a positive problem and an inverse problem [9]. The former is to calculate the edge detection signals or the change of magnetic field according to the existing electrical characteristics of the object [10], while the latter is to use the detection signals to restore the conductivity distribution map (reconstruction). Due to the soft field effect of electromagnetic field and the limitation of detector, the inverse problem has to recover more complex electrical characteristics from very few signals, which is also the key problem of MIT where underdetermination and nonlinearity coexist.

Since MIT and other electromagnetic tomography methods entered the field of vision of scholars, many reconstruction algorithms for it have been continuously proposed. They can be divided into two categories namely traditional or intelligent algorithms. The general idea of traditional ones is to discretize the imaging volume and approximate the nonlinear soft field with linear equations. The Gauss-Newton [11], Landweber iteration [12], conjugate gradient (CG) [13], Tikhonov regularization method, etc. were proposed in the early stages, and current traditional algorithms are basically their superposition or improvement. In 2015, Han min et al. [14] proposed an iterative NR algorithm based on weighted matrix and L1 norm regularization to reduce the instability of classical NR. In 2017, Liu et al. [15] proposed a novel Tikhonov regularized conjugate gradient (CG) algorithm to enhance the imaging quality. In 2019, Xiao et al. [16] reconstructed three-dimensional images of the human head model with different volumes and locations of intraaxial hemorrhaging using single-step Tikhonov regularization. Although most of these methods are relatively easy, in practical application, they can only contain limited prior information, and the calculation of an objective function gradient or Hesse matrix may be time or memory consuming [17]. Realistic needs expect more abundant prior knowledge and learn imaging features autonomously [18], to construct a complex mapping from low to high dimension.

Neural networks have even lower computational demands in dealing with nonlinear problems [19]. Some models have better real-time performance than iterative algorithms and many of them have been put forward before the popularity of deep learning. Hopfield neural network [20], radial basis function (RBF) neural network [21], and back projection network [22] have been applied earlier. In 2012, Li et al. [23] used simulation software to generate capacitance data and trained a faster RBF neural network. The effectiveness of the proposed method is verified by image reconstruction of typical flow patterns. With the popularity of convolutional neural networks (CNNs), methods based on it are applied in Electrical Impedance Tomography (EIT) image reconstruction at first [24]. In 2017, Klosowski et al. [25] proposed two reconstruction methods based on fully-connected neural network (FCNN) and CNN, respectively, which proved that CNN can be directly used between the input and the output. In 2018, Zheng et al. [26,27] developed an auto-encoder method to achieve complicated reconstruction in electrical capacitance tomography. Feng et al. [28] investigated the feasibility of a back-propagation neural network (BPNN) to reestablish the distribution of optical properties in a diffuse optical tomography (DOT) problem. Their method of evaluating resolution is also used for reference by other scholars. The stacked auto encoder (SAE) is an old method similar to one-dimensional convolution before CNN, also proved to be an effective approach to enhance images lately [29]. In 2019, Chen et al. [30] proposed an algorithm based on an SAE neural network to reconstruct the anomaly in biological tissues. Nevertheless, SAE has sensitivity to measurement, resulting in random hot pixels that appear in the images, and some scholars began to seek a breakthrough in training methods beyond the network structure. In 2020, Ren et al. [31] proposed a two-stage deep learning (TSDL) method to achieve high resolution and modeling error robust EIT image, and provided a new idea for the training mode of deep model.

Generative Adversarial Networks (GAN) uses an neural network to supervise another one. Its various variants, such as SARA-GAN and PGGAN et al., are widely used in the field of image reconstruction, including medical image enhancement [32], sample expansion [33], region of interest segmentation [34], and lesion development prediction [35]. In 2020, Liu et al. [36] used multi-stage trained GAN to reconstruct multispectral images from RGB images. The results show that GAN is helpful to solve the underdetermined problem. Yuan et al. [37] Used GAN to construct the image details in Magnetic Resonance Imaging (MRI) results in order to shorten the necessary imaging time and reduce the pain of patients. In 2021, Chen et al. [38] used generative adversarial network (GAN) to enhance MIT images calculated by complex CNN firstly, and achieved good results, yet it needs hundreds of detection signals. It should be noted that all of the above image reconstructions refer to the acquisition of higher quality images by existing images, i.e., several kinds of image to image translation process, instead of reconstructing the image directly from the detected signal. It is true that MIT measurement equipment with fewer channels can reduce the measurement cost. However, due to various limitations, the existing algorithms still cannot complete the reconstruction task based on low dimensional input well. Specifically, in the case of extremely low conductivity difference, the algorithm which only detects less than hundred electrical signals to ensure real-time performance and can clearly distinguish two anomalies has not been developed.

In view of the shortcomings of existing MIT image reconstruction methods, this paper proposed a novel MIT imaging algorithm based on generative adversarial networks. The GAN with low dimensional input was established, and trained by the data obtained from an eight-channel MIT model, where its whole training is divided into two stages, which is the improvement of the training process in this paper, referring to the TSDL method. The generator is pre-trained with its hyperparameter adjusted by back propagation with L2 regularization at first, where the problem of MIT image reconstruction was regarded as a complex nonlinear multivariate function mapping problem. Then, the generator and discriminator are combined to form a complete GAN model for joint training. Its parameters of each layer of the generator are finally separated to be used to reconstruct high-quality MIT images. Four evaluation indexes are set to compare it with BPNN and Tikhonov regularization. Experiment results show that the proposed GAN has better accuracy and robustness than others even in the case of noise. Specifically, it has smaller error, less hot pixels and artifacts, which provides a new way to solve the similar ill-posed imaging problems.

The rest of this paper is organized as follows: Section 2 presents the improved method and its principles of mathematics; Section 3 introduces the eight-channel MIT model, the process of data set building and GAN model training; Section 4 analyzes and discusses the experimental results compare with that of other algorithms; Section 5 concludes the paper, meanwhile the future works is put forward.

## 2. Methodology

### 2.1. Principles of MIT Problem

MIT is based on the principle of electromagnetic induction and eddy current testing. The relationship between the eddy current density and the electrical characteristics of imaging object is described as Equation (1) according to Maxwell equations as follows:

(1) $$J_{e}=\left(\sigma +j\omega \varepsilon \right)E$$

where eddy current $J_{e}$ is composed of two parts, ε is the permittivity that can be ignored in the inverse problem and eddy current is mainly generated by the conductivity σ in the biological object, with physical modeling and finite element model (FEM) discretization, the deterministic MIT measurement model can be expressed as:

(2) $$V=F\left(\sigma \right)$$

where V is a vector of the measured phase of boundary voltage, F is the forward model map of the internal conductivity distribution, which is usually linearized to the sensitivity matrix in traditional algorithms as V=Sσ. The purpose of the MIT image reconstruction (an ill-posed, nonlinear inverse problem) is to obtain the conductivity distributions by solving the model in Equation (2). The inverse problem is generally expressed as $\sigma =F^{-1}\left(V\right)$ or $\sigma =S^{-1}V$.

Furthermore, the sensitivity matrix cannot accurately describe the corresponding relationship between the measured value and conductivity due to the soft field effect of electromagnetic field, which increases the difficulty of solving MIT problem. This is also a reason why the traditional algorithms are not always satisfactory.

### 2.2. Principles of ANN

Artificial neural network is a kind of mathematical structure formed by amounts of data processing units (or neurons) connected by directional operations. It is an digital abstraction of the mechanism of the brain. Specifically, each neuron represents a mapping relation called activation function. Connections between neurons represent the weights for signal passing through these connections, equivalent to network memory. The output of an ANN varies according to its different connection structures, weight values, and activation functions. This kind of output mode is called forward propagation in studies, as shown below:

(3) $$a^{\left(l+1\right)}=f\left(W_{l+1,l}a^{\left(l\right)}+b^{\left(l\right)}\right)$$

where $a^{\left(i\right)}$ represents the values of neurons in layer i, and f represents the activation function of this layer. W and b are the weights and bias (matrix) to be optimized, collectively referred to as parameters matrix θ, and ANNs are able to fit various complex nonlinear functions by modifying θ. With the deepening of layers, they can describe increasingly complex mapping relationships.

Back propagation neural network (BPNN) is a fully connected ANN trained by error back propagation algorithm, which is based on the chain derivative rule. It transfers the error between the predicted result and the real value along the opposite direction of output mode, so as to adjust parameters matrix and optimize the fitting effect. It has the advantages of being simple and fast, with less super parameters, easy training, and convergence. The following GAN uses two such networks with different layers to achieve the goal of image reconstruction.

### 2.3. The GAN for MIT Image Reconstruction

Generative adversarial network (GAN) is a class of machine learning frameworks. Two neural networks contest with each other in a game (in the form of a zero-sum game). This technique learns to generate new data with the same statistics as the training set. Its key idea is based on the “indirect” training through the discriminator, which basically means that the generator is not only trained to minimize the distance to a specific image, but also to fool the discriminator, similar to supervised maximum likelihood estimation. The underdetermined and non-linear problems can be solved by it, and the over-fitting caused by direct feedback can be avoided.

**Figure 1:**
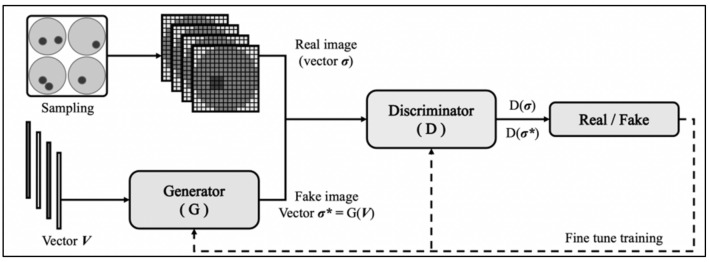
The training process of MIT-GAN model.

To build a GAN model for an MIT problem, this paper pre-trains the generator firstly using L2 regularization, takes phase difference as input, adjusts the network architecture to complete simple imaging tasks, then connects it with a discriminator to form a counter network, and adds a cross verification set to monitor its convergence. When the generator error is less than the discriminator error and the verification set error is less than 0.05, the training is stopped. The general training process is shown in Figure 1. Its main mathematical logic will be introduced next.

The essence of the generator model here is a maximum likelihood estimate, which maximizes the likelihood of conductivity reconstructed by the generator based on the true distribution.

(4) $$L\left(G,V,\sigma \right)=\prod_{i=1}^{M}P_{G\left(V\right)}\left(\sigma^{\left(i\right)};\theta_{g}\right)$$

where the target is to find the appropriate generator parameter $\theta_{g}^{*}$, where g represents the generator, V represents the voltage, σ represents the conductivity, and P represents the discrete distribution function.

(5) $$\theta_{g}^{*}=\underset{\theta_{g}}{\mathrm{argmax}}\prod_{i=1}^{M}P_{G\left(V\right)}\left(\sigma^{\left(i\right)};\theta_{g}\right)\;\Theta_{g}^{*}=\underset{\theta_{g}}{\mathrm{argmax}}\log\prod_{i=1}^{M}P_{G\left(V\right)}\left(\sigma^{\left(i\right)};\theta_{g}\right)\;\Theta_{g}^{*}=\underset{\theta_{g}}{\mathrm{argmax}}\sum_{i=1}^{m}\log P_{G\left(V\right)}\left(\sigma^{\left(i\right)};\theta_{g}\right)\;\Theta_{g}^{*}=\underset{\theta_{g}}{\mathrm{argmax}}\;E_{\sigma }[\log P_{G\left(V\right)}\left(\sigma^{\left(i\right)};\theta_{g}\right)]\;\Theta_{g}^{*}=\underset{\theta_{g}}{\mathrm{argmax}}\int_{\sigma }^{\;}p_{\sigma }\left(x\right)\log p_{G\left(V\right)}\left(x;\theta_{g}\right)\mathrm{dx}-\int_{\sigma }^{\;}p_{\sigma }\left(x\right)\log p_{\sigma }\left(x\right)\mathrm{dx}$$

where E represents the mathematical expectation and p represents the continuous distribution function. The term $\int_{\sigma }^{\;}p_{\sigma }\left(x\right)\log p_{\sigma }\left(x\right)\mathrm{dx}$ subtracted from the above formula, which is independent of $\theta_{g}$, shall not affect the solution of the maximum point. The purpose is to construct the following Kullback–Leibler divergence, a measure of probability distribution similarity in statistics.

(6) $$\theta_{g}^{*}=\underset{\theta_{g}}{\mathrm{argmax}}\int_{\sigma }^{\;}p_{\sigma }\left(x\right)\log\frac{p\left(x;\theta_{g}\right)}{p_{\sigma }\left(x\right)}\mathrm{dx}\;\Theta_{g}^{*}=\underset{\theta_{g}}{\mathrm{argmin}}\;\mathrm{KL}\left(P_{\sigma }\left(x\right)∥P_{G\left(V\right)}\left(x;\theta_{g}\right)\right)$$

This K–L divergence minimization problem cannot be solved directly with maximum likelihood, so the discriminator loss is defined as follows to optimize the generator parameters, that is, to calculate instead of maximum likelihood estimation:

(7) $$\mathrm{Value}\left(D,G\right)=\int_{\sigma }^{\;}p_{\sigma }\left(x\right)\log D\left(x\right)\mathrm{dx}+\int_{V}^{\;}p_{V}\left(x\right)\log\left(1-D\left(G\left(x\right)\right)\right)\mathrm{dx}$$

According to the Radon–Nikodym theorem:

(8) $$\mathrm{Value}\left(D,G\right)=\int_{\sigma }^{\;}(p_{\sigma }\left(x\right)\log D\left(x\right)+p_{G}\left(x\right)\log\left(1-D\left(x\right)\right))\mathrm{dx}$$

where $p_{\sigma }\left(x\right)$ and $p_{G}\left(x\right)$ are independent of the discriminator $D\left(x\right)$. Let the integrand of the above formula take the derivative of $D\left(x\right)$ and make it equal to 0:

(9) $$\frac{p_{\sigma }\left(x\right)}{D\left(x\right)}+\frac{p_{G}\left(x\right)}{D\left(x\right)-1}=0\Rightarrow D\left(x\right)=\frac{p_{\sigma }\left(x\right)}{p_{\sigma }\left(x\right)+p_{G}\left(x\right)}$$

It can be proved that if and only if $p_{\sigma }\left(x\right)=p_{G}\left(x\right)$, $D\left(x\right)=0.5$, which is the only optimal solution to this problem, means the reconstructed conductivity calculated by the generator is exactly consistent with the real conductivity so that the discriminator can’t tell whether the image is from generator or real samples. Independent back-propagation procedures are applied to both networks so that the generator produces better images while the discriminator becomes more skilled at flagging synthetic images. The generator and discriminator of GAN used in this paper are both BPNN to adapt to voltage and conductivity vector. The objective function for this GAN model is:

(10) $$\underset{\theta_{g}}{\min}\;\underset{\theta_{d}}{\max}\;\mathrm{Value}\left(D,G\right)=E_{\sigma }\left[\mathrm{logD}\left(\sigma \right)]+E_{V}[\log\left(1-D\left(G\left(V\right)\right)\right)\right]$$

Due to the co-evolution of generators and discriminators, it performs better than general regularization or supervised training algorithms and are less prone to fall into local optimum. The optimization process is as following Algorithm 1.
**Algorithm 1** Gradient descent optimization of GAN**for** number of training iterations **do** **for** k steps **do** Sample batch of $M_{num}$ samples from voltage distributions $\left\{V^{\left(1\right)},\ldots ,V^{\left(M\right)}\right\}$. Sample batch of $M_{num}$ samples from conductivity distributions $\left\{\sigma^{\left(1\right)},\ldots ,\sigma^{\left(M\right)}\right\}$. Update the discriminator by ascending its stochastic gradient: $\nabla_{\theta_{d}}\frac{1}{m}\sum_{i=1}^{M_{num}}\left[\mathrm{logD}\left(\sigma^{\left(i\right)}\right)+\log\left(1-D\left(G\left(V^{\left(i\right)}\right)\right)\right)\right]$. **end for** Sample batch of $M_{num}$ samples from voltage distributions $\left\{V^{\left(1\right)},\ldots ,V^{\left(M\right)}\right\}$. Update the generator by descending its stochastic gradient: $\nabla_{\theta_{g}}\frac{1}{m}\sum_{i=1}^{M_{num}}\log\left(1-D\left(G\left(V^{\left(i\right)}\right)\right)\right)$. **end for**

### 2.4. Data Set Preparing and Network Training

The needed data sets are obtained by solving the MIT forward problem with finite element method before the training. Each sample consists of two parts: the conductivity distribution of the sampling grid and the computed phase difference of the measured boundary voltage. The process of obtaining experimental samples is shown in Algorithms 2 and 3.
**Algorithm 2** Construct training samplesThe sampling grid and 8-coil MIT model are established. **for** number of needed samples **do** Load the model. **for** $\left\{\rho^{\left(i\right)},\theta^{\left(i\right)}\right\}$ in coordinates of anomalies **do** **for** j in coordinates of number of coils (8) anomalies **do** Assign $\left\{\rho^{\left(i\right)},\theta^{\left(i\right)}\right\}$ to change the position of anomaly (anomalies) in the model. The *j*-th coil acts as the excitation and the others as the receiver. Re geometric modeling and mesh generation. Run the model. 7 voltage data are obtained from the receiving coils. 3409 conductivity data are obtained from the sampling grid. **end for** **end for** Vec-operator the collected voltage data and the conductivity data. Save as a corresponding sample $\left\{V^{\left(num\right)},\sigma^{\left(num\right)}\right\}$. **end for**
**Algorithm 3** Construct training samplesLoad the exiting dataset. **for** number of expansion times **do** Build a square lattice same with sampling grid. **for** $\left\{\rho^{\left(i\right)},\theta^{\left(i\right)}\right\}$ and $\left\{V^{\left(i\right)},\sigma^{\left(i\right)}\right\}$ in i-th sample **do** Convert polar coordinate $\left\{\rho^{\left(i\right)},\theta^{\left(i\right)}+num•45^{{}^{\circ}}\right\}$ to rectangular coordinate $\left\{x^{\left(i\right)},y^{\left(i\right)}\right\}$. Set points $\left(x,y\right)$ meets ${\left(x-x^{\left(i\right)}\right)}^{2}+{\left(y-y^{\left(i\right)}\right)}^{2}<{\mathrm{radius}}^{2}$ in square lattice as anomalies. Read the conductivity signal $\sigma^{\left(j\right)}$ in sequence again. Move the data in $V^{\left(i\right)}$ forward 7 units in sequence to form $V^{\left(j\right)}$. **end for** Save these $\left\{V^{\left(1\right)},\sigma^{\left(1\right)},\ldots ,V^{\left(j\right)},\sigma^{\left(j\right)},\ldots ,V^{\left(num\right)},\sigma^{\left(num\right)}\right\}$ as corresponding new samples. **end for**

Firstly, the generator is pre-trained, and then it is connected to the discriminator for GAN training. Having obtained the voltages $\left\{V^{\left(1\right)},V^{\left(2\right)},\ldots ,V^{\left(M\right)}\right\}$ with conductivities $\left\{\sigma^{\left(1\right)},\sigma^{\left(2\right)},\ldots ,\sigma^{\left(M\right)}\right\}$ where M is the number of training samples, $V^{\left(k\right)}\in {\left[0,1\right]}^{n}$ and $\sigma^{\left(k\right)}\in {\left[0,1\right]}^{m}$ (m is the number of phase differences of each detected sequence while n equals to the number of pixel of conductivity images), the BPNN denoted by $G_{\theta }$ can be trained several epochs. In the first stage, given the output ${\hat{\sigma }}^{\left(i\right)}=G_{\theta }\left(V^{\left(i\right)}\right)$, we seek to minimize L2-error between that to real conductivity vector:

(11) $$\mathrm{Loss}\left(\sigma ,\hat{\sigma }\right)={∥\sigma -\hat{\sigma }∥}_{2}^{2}$$

The above purpose is to determine the hyperparameters of BPNN as a generator to improve the performance in the subsequent training of GAN. In the second stage, the pre-trained network is as the initialization of the generator. Its outputs are as the inputs of discriminator with real conductivity distributions as labels. According to activation function of all layers, the gradient descent algorithm, based on the Adam optimizer, adjusts every parameter as follows:

(12) $$\theta ≔\theta -\alpha \cdot \nabla \mathrm{Loss}\left(\theta \right)$$

where α is learning rate. After hyperparameters selection finished, a needed cross-validation set as $\left\{\left(V^{\left(1\right)},\sigma^{\left(1\right)}),\left(V^{\left(2\right)},\sigma^{\left(2\right)}\right),\ldots ,(V^{\left(N\right)},\sigma^{\left(N\right)}\right)\right\}$, a small number of untrained samples selected from the total data set, is used for supervised learning. Because the generator and discriminator of the GAN confront each other, which leads to the instability of their gradient descent and have to be monitored to determine the conditions of cessation in real time. A needed stronger generator can be selected when cross-validation signified its loss are smaller. The effect of cross validation is evaluated by MSE as follows:

(13) $$\mathrm{MSE}\left(y,\hat{y}\right)=\frac{1}{m}\sum_{i=1}^{m}{\left(y_{i}-{\hat{y}}_{i}\right)}^{2}$$

which is the form of vector L2 error averaging to each dimension.

## 3. Experiments

### 3.1. MIT Signal Detection Model

**Figure 2:**
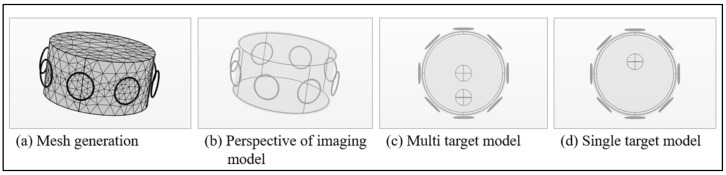
The model of 8-channel MIT signal acquisition system.

In this paper, an 8-channel MIT measurement model was established with an anomaly placed in the circular imaging range with 8 coils around. After the excitation current was applied to one of these coils, the induced voltage of other detection coils was extracted. The general structure is shown in Figure 2.

The cylinder in the model represents the measured target tissue, with a height and a radius of 0.1 m, which is also the imaging radius. Eight identical 30 turn coils with a radius of 0.025 m were fixed on the surface circle of its longitudinal center, and their centers were 0.11 m away from the tissue center. Meanwhile, the eight coils were numbered counterclockwise, from coil 1 to coil 8. The conductivity of anomaly was 0.05 S/m to simulate the electrical characteristics of hematoma, and that of the cylinder model is 0.25 S/m to simulate the electrical characteristics of normal tissues. The alternating current with root mean square of 1 A and frequency of 10 MHz was introduced into each coil and received by other coils, a voltage vector with dimension of 56 can be measured in turn. At the same time, conductivity values with equal interval distribution can be extracted according to the dense square array within the circular range of imaging as the standard value of post reconstruction evaluation.

### 3.2. Data Set Preparation

In order to prepare for the optimization of artificial neural network model, needed data sets have to be generated for its training and testing. A widely used method was to generate samples with random parameters. To solve the inherent disadvantage of the MIT inverse problem, the poor sensitivity in the central region of imaging volume, the sample generation method moves the anomaly according to the polar coordinates, generate samples in turn, and then shuffle the order. In samples of a single target, a sphere anomaly with a radius of 2 cm was used to move from the center to the edge of the imaging region in turn. The angle step of each movement was 9°, and the radial step was 0.1 cm until the anomaly intersects the imaging boundary. The approximate method was also used in samples with two anomalies. Then, more samples were copied by rotating and symmetry. The measured voltage vector V and conductivity vector σ were stored as a matrix. The inputs correspond to the outputs, and they were scaled to the range of 0 to 1, which is normalization in deep learning. A total of 36,540 sets of data were obtained, including 2840 single-target and 33,664 two-anomaly samples. The following experiments were based on this identical database. The data set is generated by COMSOL multiphysics 5.5 with MATLAB.

### 3.3. Training and Reconstruction

**Figure 3:**
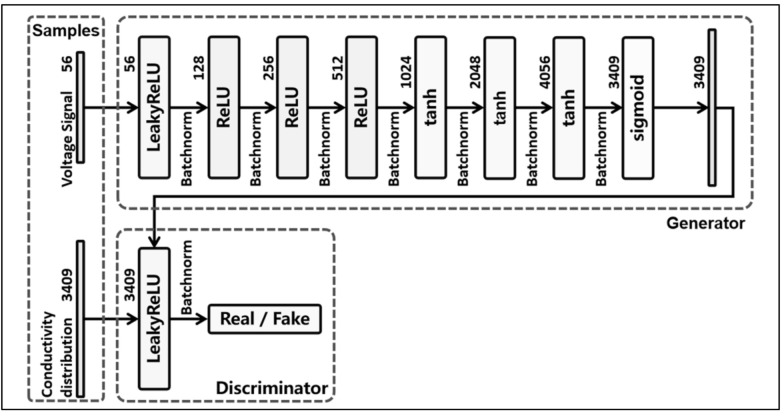
The hyperparameters and activation functions of GAN. The voltage data is input into the generator of the 8-layer structure, its results are compared with the real conductivity distribution by the discriminator.

Data sets were divided into two parts for training and testing. They were input into the GAN network as shown in Figure 1. One thousand epochs of the generator were trained to reduce loss using the Adam optimizer. The number of iterations and the learning rate have a serious effect on the accuracy of a DNN model. To minimize its loss, the generator structure of the proposed GAN was finally {56 128 256 512 1024 2048 4096 3409}. The activation function of its first four layers was ReLU, the fifth to seventh layers were tanh, and the last layer was sigmoid to satisfy the classification output between 0 and 1 (Figure 3).

Other hyperparameters were set as follows: In the pre-training phase of generator, the epoch was 1000 and the batch-size was 50. In the GAN fine-tuning phase, the batch-size was equal to 4000. In addition, control experiments were designed based on the same data set, including the classical and other intelligent algorithms.

In this paper, the image is reconstructed with 3409 pixels in a circular object field, with a 67 × 67 square grid. Algorithmic programming and image reconstruction were implemented under the Python 3.6 with Tensorflow 1.14.0 on a PC configured for 2.50 GHz CPU, 12.0 GB of memory.

## 4. Results and Discussion

### 4.1. Evaluating Indicators

Based on the requirements of image reconstruction, the following five evaluation indexes are proposed to measure the effect of model reconstruction.

#### 4.1.1. Root Mean Squared Error (RMSE)

The sum of the square of the deviation between the reconstructed value, the true value, and the square root of the ratio of the total conductivity of the reconstructed sample m, which is used to measure the deviation between the reconstructed conductivity value and the true value. The RMSE is calculated as follows:

(14) $$\mathrm{RMSE}\left(y,\hat{y}\right)=\sqrt{\frac{1}{m}\sum_{i=1}^{m}{\left(y_{i}-{\hat{y}}_{i}\right)}^{2}}$$

#### 4.1.2. Structural Similarity (SSIM)

Structural similarity is an index to measure the similarity of two images. SSIM uses two images, one is an uncompressed image without distortion, and the other is a distorted image. It is here used to evaluate the degree of image distortion after reconstruction:

(15) $$\mathrm{SSIM}\left(y,\hat{y}\right)=\frac{\left(2\mu_{y}\mu_{\hat{y}}+c_{1}\right)\left(2\sigma_{y\hat{y}}+c_{2}\right)}{\left(\mu_{y}^{2}+\mu_{\hat{y}}^{2}+c_{1}\right)\left(\sigma_{y}^{2}+\sigma_{\hat{y}}^{2}+c_{2}\right)}$$

where μ and $\sigma^{2}$ represent mean and variance, and $\sigma_{y\hat{y}}$ represents covariance. $c_{1}$ and $c_{2}$ are constants and are calculated as:

(16) $$c_{1}={\left(k_{1}L\right)}^{2}$$

(17) $$c_{2}={\left(k_{2}L\right)}^{2}$$

where $k_{1}$ is equal to 0.01 and $k_{2}$ is equal to 0.03. L is the range of the output value.

#### 4.1.3. Peak Signal to Noise Ratio (PSNR)

Peak signal to noise ratio is the most common and widely used objective measure to assess picture quality, avoiding the subjective impact of direct observation (e.g., the human eye is more sensitive to differential contrast with lower spatial frequency). The PSNR is calculated as follows:

(18) $$\mathrm{PSNR}\left(y,\hat{y}\right)=10\log_{10}\frac{{\left(2^{n}-1\right)}^{2}}{\mathrm{MSE}\left(y,\hat{y}\right)}$$

where n is the binary digit of the image and MSE is the mean squared error (Equation (13)).

#### 4.1.4. Correlation Coefficient (CC)

A correlation coefficient is a numerical measure of some type of correlation, meaning a statistical relationship between two variables. Several types of correlation coefficient exist, each with their own definition and own range of usability and characteristics. Here, we use:

(19) $$\mathrm{CC}\left(y,\hat{y}\right)=\frac{\sigma_{y\hat{y}}}{\sqrt{\sigma_{y}\sigma_{\hat{y}}}}$$

It assumes values in the range from −1 to +1, where ±1 indicates the strongest possible agreement and 0 the strongest possible disagreement.

### 4.2. Results and Analysis

Here we give the average value of 500 newly generated random test samples under four evaluation indices, including that with the different amplitude noises added, which is measured by the signal-to-noise ratio (SNR).

**Figure 4:**
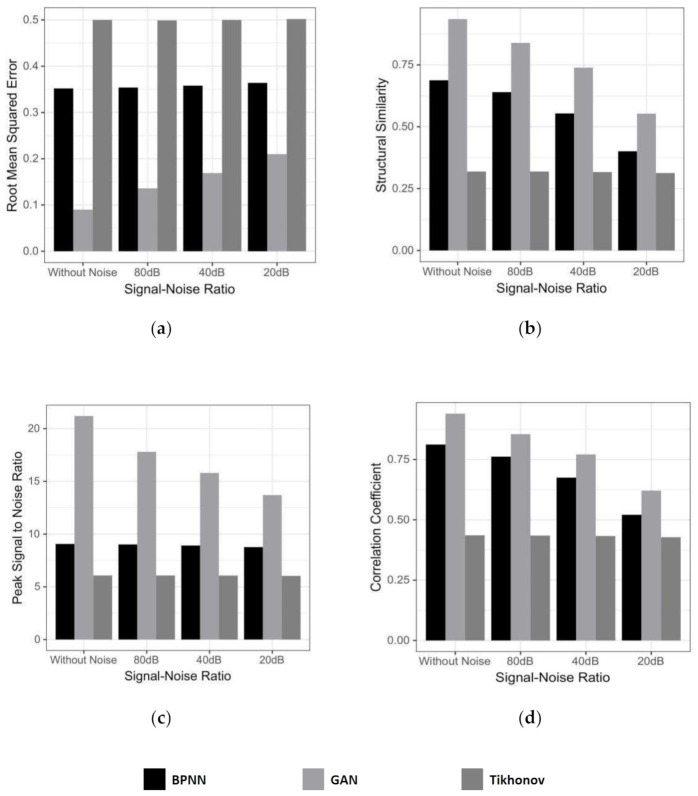
The average value of evaluating indicators, under different algorithms and SNR, as follows: (**a**) RMSE, (**b**) SSIM, (**c**) PSNR, and (**d**) CC.

With the increase of noise amplitude, the index change of each model is shown in Figure 4. Figure 4a means that their reconstruction effect is worse as the noise increases. The RMSE of GAN increases from 0.1 to 0.2, more than doubled. In contrast, Tikhonov and BPNN are much more stable, about 0.5 and 0.35, respectively, but both are larger than GAN. Figure 4b illustrates an SSIM comparison, GAN is also far ahead, reaching more than 0.9 when noiseless. Figure 4c shows the effect of noise on PSNR. Its value range of GAN is (13, 22). Tikhonov and BPNN are stable at about 6 and 8, respectively. However, the BPNN without noise is not even as good as GAN with SNR of 20 dB. This signifies that GAN can filter noises in the signal as much as possible to avoid mapping to the image. Figure 4d shows that all three reconstruction methods can make the image positively correlated with the true value. The GAN algorithm is the best, with a mean up to (0.6, 0.9). What makes a discriminator superior to regularization is that it is only responsible for avoiding poor reconstruction results rather than a restriction on the range of parameter values.

**Figure 5:**
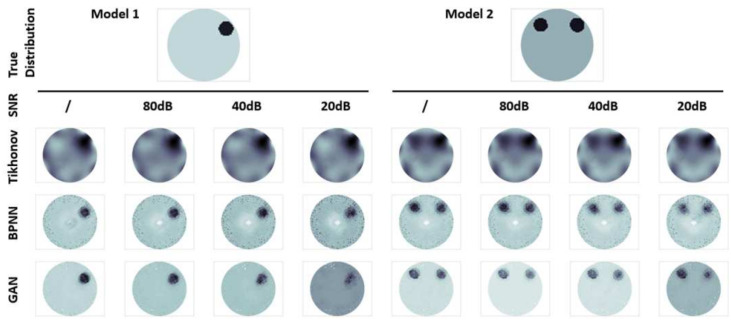
The image reconstruction under different noise and algorithm.

**Figure 6:**
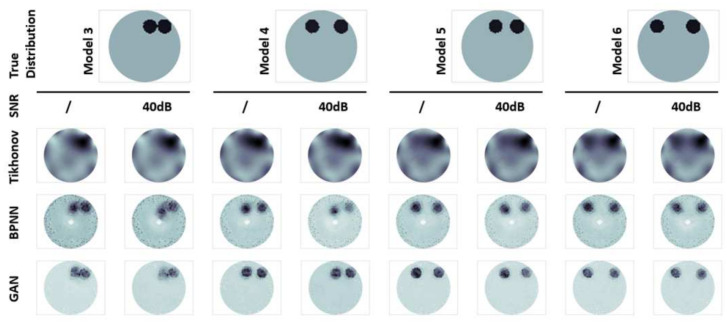
The image reconstruction under different noise and algorithm.

In order to intuitively prove and reflect their advantages and disadvantages in robustness and resolution, we also give comparative experiments of imaging state and evaluation index of representative samples under different noise and different algorithms. In addition to the noiseless state, the SNR is 80, 40, and 20 dB, respectively; the minimum resolution distances of the two anomalies are set as 0, R, 2R, and 3R, respectively, where R is the radius of the anomalies. The pictures are shown in Figure 5 and Figure 6, and the relevant data are listed in Table 1, Table 2, Table 3 and Table 4.

It can be seen from the figures that although the influence of noise on GAN is slightly greater than other models, it is still better than BPNN and Tikhonov at any SNR. It also performs well in distinguishing two anomalies even they are tight together. In contrast, the BPNN model has more noise, while Tikhonov regularized image has a large area of artifacts. This also confirms the fact that GAN can filter noises.

From the data in the table, it appears that GAN exceeds 0.8 in both the SSIM and CC, which almost perfectly represents the true distribution of conductivity. RMSE can also be reduced below 0.2, although slightly higher in multi-target recognition, it is still satisfactory. From the point of view of signal restoration, its SSIM is over 15, which is much better than the other two algorithms.

## 5. Conclusions and Future Works

According to the basic principle of image reconstruction for MIT and the basic ideal of artificial neural network training, an MIT image reconstruction algorithm based on GAN is proposed in this paper. Firstly, an eight-coil MIT detection model is built, and anomalies in different positions are added. COMSOL Multiphysics software is used to solve the forward problem, and 36,540 of training samples are achieved after expansion. Secondly, the GAN model is built, and its generator is pre-trained by data sets with L2 regularization, and the network that can initially complete the reconstruction task is obtained. Then the generator and the discriminator are connected for joint training, and the convergence is determined by the cross-validation set. The separated generator can reconstruct the high quality conductivity distribution image according to the voltage signal. Finally, the reconstruction results of 500 new models that have not been used for training show that the average root mean square error of the proposed method is 0.090 and the average correlation coefficient is 0.940, which is much better than the traditional reconstruction algorithm Tikhonov regularization and BPNN using L2 regularization. This method provides a more effective solution for ill-posed inverse problem in imaging field.

Moreover, on this basis we can try to expand the imaging range to three-dimensional when collecting the conductivity signal. Specifically, it is not only to establish the sampling grid on the plane with z = 0, but also to divide several layers equidistant in the z direction, for example, to represent a 3D image by stacking 10 2D images in sequence. In this case, the structure of the artificial neural network should be adjusted accordingly, such as changing the vector output to the matrix output. It is also likely that the training time and even the computer hardware system put forward higher requirements, so that this algorithm still has potentials for further imaging studies.

## Figures and Tables

**Table 1:** The RMSE of two models under different noise and algorithm.

Model	Model 1				Model 2			
SNR	/	80 dB	40 dB	20 dB	/	80 dB	40 dB	20 dB
Tikhonov	0.4728	0.4800	0.4848	0.4758	0.4572	0.4590	0.4547	0.4523
BPNN	0.3358	0.3335	0.3393	0.3354	0.3501	0.3498	0.3520	0.3521
GAN	0.0753	0.0939	0.1075	0.1296	0.1706	0.1776	0.1681	0.1726

**Table 2:** The SSIM of two models under different noise and algorithm.

Model	Model 1				Model 2			
SNR	/	80 dB	40 dB	20 dB	/	80 dB	40 dB	20 dB
Tikhonov	0.4641	0.4547	0.4579	0.4482	0.4386	0.4590	0.4455	0.4500
BPNN	0.6751	0.6432	0.5967	0.5147	0.7029	0.6888	0.6430	0.5163
GAN	0.9216	0.8847	0.8356	0.7311	0.8517	0.8404	0.8427	0.8143

**Table 3:** The PSNR of two models under different noise and algorithm.

Model	Model 1				Model 2			
SNR	/	80 dB	40 dB	20 dB	/	80 dB	40 dB	20 dB
Tikhonov	6.5061	6.3752	6.2879	6.4513	6.7987	6.7631	6.8461	6.8896
BPNN	9.4776	9.5376	9.3876	9.4894	9.1161	9.1244	9.0698	9.0658
GAN	22.4598	20.5428	19.3657	17.7510	15.3569	15.0083	15.4862	15.2568

**Table 4:** The CC of two models under different noise and algorithm.

Model	Model 1				Model 2			
SNR	/	80 dB	40 dB	20 dB	/	80 dB	40 dB	20 dB
Tikhonov	0.5770	0.5699	0.5858	0.5538	0.5819	0.5785	0.5899	0.5890
BPNN	0.7974	0.7581	0.7064	0.6542	0.8276	0.8160	0.7708	0.6663
GAN	0.9254	0.8842	0.8410	0.7576	0.8807	0.8703	0.8586	0.8180

## Data Availability

Not applicable.
